# Supplementary material for: Exploring the Role of Platelets in Virus-Induced Inflammatory Demyelinating Disease and Myocarditis
Source: Int J Mol Sci. 2024 Mar 19;25(6):3460. doi: 10.3390/ijms25063460 (PMC10970283; doi:10.3390/ijms25063460)
Supplement: Supplementary file 1 [file ijms-25-03460-s001.zip › Supplemental Material Figures S1-S Table S1.pdf]

Supplemental Materials

Exploring the Role of Platelets in Virus-Induced Inflammatory Demyelinating Disease and Myocarditis

Ijaz Ahmad[1†], Seiichi Omura[1†], Fumitaka Sato[1], Ah-Mee Park[1,2], Sundar Khadka[1,3], Felicity N. E. Gavins[4], Hiroki Tanaka[5], Motoko Y. Kimura[6], and Ikuo Tsunoda[1*]

[1]Department of Microbiology, [2]Department of Arts and Science, Kindai University Faculty of Medicine, Osaka, Japan

[3]Department of Immunology, Duke University, North Carolina, USA

[4] Department of Biosciences, Centre for Inflammation Research and Translational Medicine, College of Health and Life Sciences, Brunel University London, Uxbridge, UK

[5]Division of Tumor Pathology, Department of Pathology, Asahikawa Medical University, Asahikawa, Japan

[6] Department of Experimental Immunology, Graduate School of Medicine, Chiba University, Chiba, Japan

[†]These authors contributed equally to this work.

***Correspondence:**

Ikuo Tsunoda, M.D., Ph.D.

Department of Microbiology, Kindai University Faculty of Medicine

377-2 Ohnohigashi, Osakasayama, Osaka 589-8511 Japan

E-mail: itsunoda@med.kindai.ac.jp

Table of contents

Supplemental Figure S1. Validation of RNA-seq transcription data by real-time PCR 3

Supplemental Figure S2. Quantification of viral genome in the transcriptome data of the spinal cord and platelets in Theiler's murine encephalomyelitis virus (TMEV) infection 4

Supplemental Figure S3. Principal component analysis (PCA) of the splenic transcriptome data in TMEV infection 5

Supplemental Figure S4. PCA of splenic transcriptome data from age-matched control mice 6

Supplemental Figure S5. PCA of platelet transcriptome data of all TMEV-infected and control samples 7

Supplemental Figure S6. Platelet detection in the central nervous system (CNS) and heart of TMEV infection and experimental autoimmune encephalitis (EAE) 8

Supplemental Figure S7. Effects of a neutralizing antibody against platelets on mice 9

Supplemental Figure S8. Lymphoproliferative responses to TMEV 10

Supplemental Table S1. Upregulations of platelet-related genes in TMEV infection and EAE 11

Supplemental Table S2. Database for annotation, visualization, and integrated discovery (DAVID) functional clustering of differential expressed genes (DEGs) ($P < 0.05$) in the spleen of TMEV-infected mice days 4 (S2A), 7 (S2B), and 35 (S2C) post-infection (p.i.) ..... Supplemental Table S2.xlsx

Supplemental Table S3. Lists of genes in each cluster of *k*-means clustering of the splenic transcriptome data ..... Supplemental Table S3.xlsx

Supplemental Table S4. DAVID functional clustering of genes in each cluster of the splenic transcriptome of TMEV-infected mice ..... Supplemental Table S4.xlsx

Supplemental Table S5. DAVID functional clustering of DEGs ($P < 0.05$) in platelets of TMEV-infected mice days 4 (S5A), 7 (S5B), and 35 (S5C) p.i. ..... Supplemental Table S5.xlsx

Supplemental Table S6. Lists of genes in each cluster of *k*-means clustering of the platelet transcriptome data ..... Supplemental Table S6.xlsx

Supplemental Table S7. DAVID functional clustering of genes in each cluster of the platelet transcriptome of TMEV-infected mice ..... Supplemental Table S7.xlsx

Accession no. GSE253385, URL: https://www.ncbi.nlm.nih.gov/geo/query/acc.cgi?acc=GSE253385

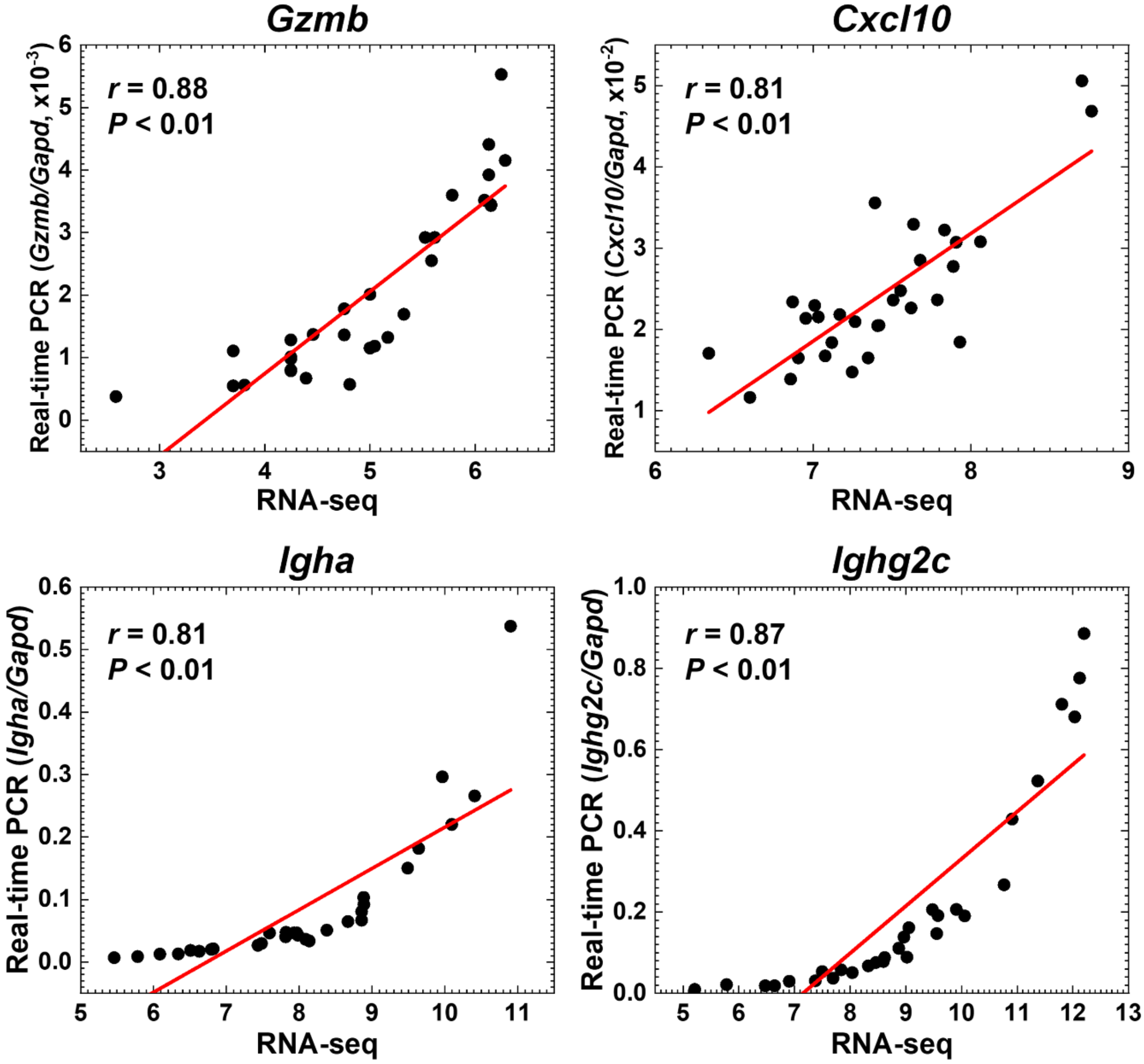

Supplemental Figure S1. Validation of RNA-seq transcriptome data by real-time PCR. To validate a method of transcriptome data analysis, we conducted real-time PCR of four immune genes [granzyme B (*Gzmb*), C-X-C motif chemokine ligand 10 (*Cxcl10*), immunoglobulin heavy constant alpha (*Igha*), and immunoglobulin heavy constant gamma 2C (*Ighg2c*)], using spleen samples. We confirmed that the transcriptome data were correlated with real-time PCR data.

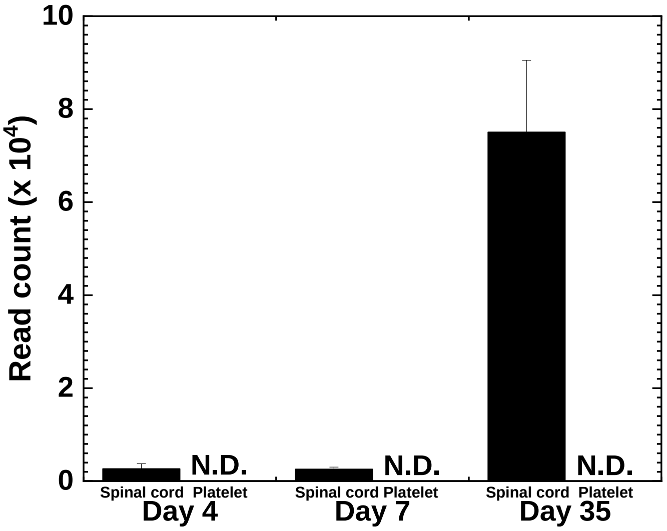

Supplemental Figure S2. Quantification of viral genome using the transcriptome data of spinal cord and platelet samples in Theiler murine encephalomyelitis virus (TMEV) infection. We infected SJL/J mice with TMEV and harvested the spinal cord and platelets 4, 7, and 35 days post-infection (p.i.). In the spinal cord, viral genome was detected throughout the disease course and increased 35 days p.i. On the other hand, in the platelets, viral genome was not detected at any time points. Five to seven mice per group. N.D.; not detected.

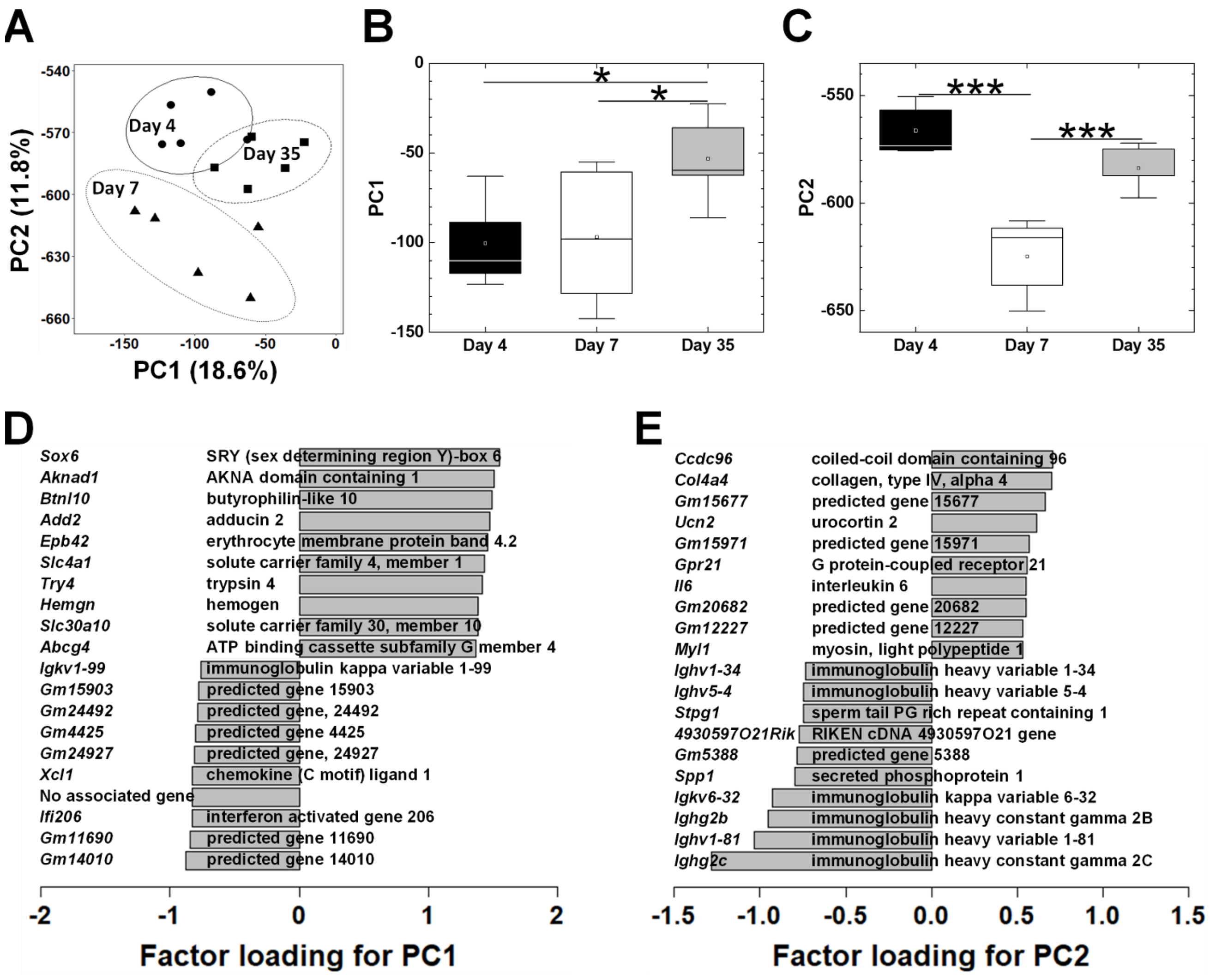

Supplemental Figure S3. Principal component analysis (PCA) of the splenic transcriptome data in TMEV infection. We infected SJL/J mice with TMEV and harvested the spleen samples 4, 7, and 35 days p.i. **(A)** We conducted PCA using the splenic transcriptome data 4 (●), 7 (▲), and 35 (■) days p.i. PCA separated the samples into three groups which were consistent with the three time points. The values in parenthesis indicated the proportion of variance of each principal component (PC). **(B)** PC1 values were significantly higher 35 days p.i. than 4 and 7 days p.i. [*, $P < 0.05$, analysis of variance (ANOVA)]. **(C)** PC2 values were significantly lower 7 days p.i. than 4 and 35 days p.i. (***, $P < 0.001$, ANOVA). **(D, E)** Factor loading for PC1 and PC2 showed the top 10 genes correlating with PC1 and PC2 values positively and negatively. In the boxplots, the open square, middle line, box, lower whisker, and upper whisker indicate the mean, median, interquartile range, minimum, and maximum. The total sample number was five per group.

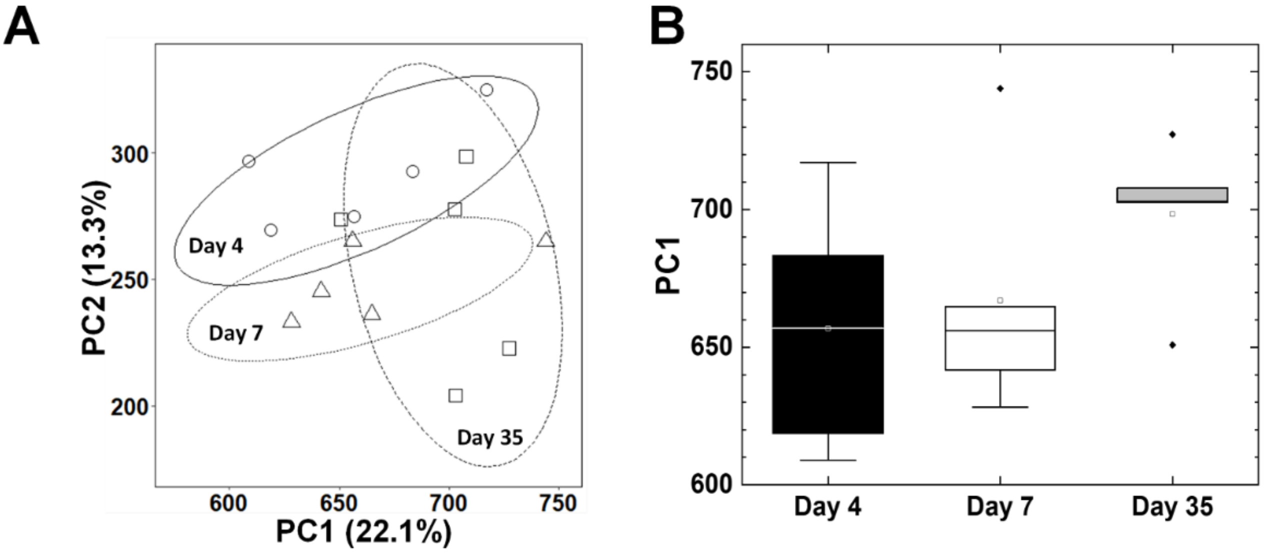

Supplemental Figure S4. PCA of the splenic transcriptome data from age-matched uninfected control mice on days 4 (○), 7(△), and 35 (□). **(A)** PCA did not separate the samples into distinct groups. The values in parenthesis indicated the proportion of variance of each PC. **(B)** PC1 values showed no statistical differences among the three time points. In the boxplots, the open square, middle line, box, lower whisker, upper whisker, and closed rhombus indicate the mean, median, interquartile range, minimum, maximum, and outlier. The total sample number was five per group.

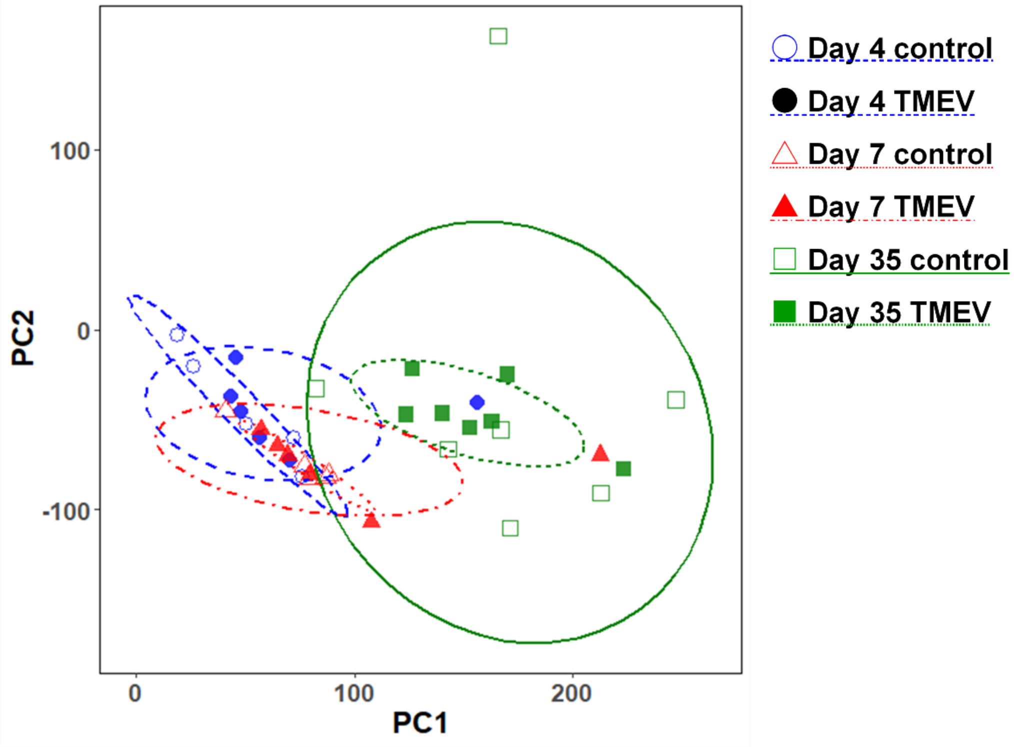

Supplemental Figure S5. PCA of all platelet transcriptome data on days 4, 7, and 35 from TMEV-infected mice and control mice. PCA did not separate the samples between the control and TMEV-infected groups at any time points. On the other hand, PCA separated the samples into two groups: one group was composed of samples 4 and 7 days of TMEV-infected and control mice, and the other was composed of samples 35 days of TMEV-infected and control mice. This separation likely reflected maturation-related gene expression changes in platelets. The symbols showed control mice on days 4 (○), 7(△), and 35 (□) and TMEV-infected mice on days 4 (●), 7 (▲), and 35 (■) p.i.

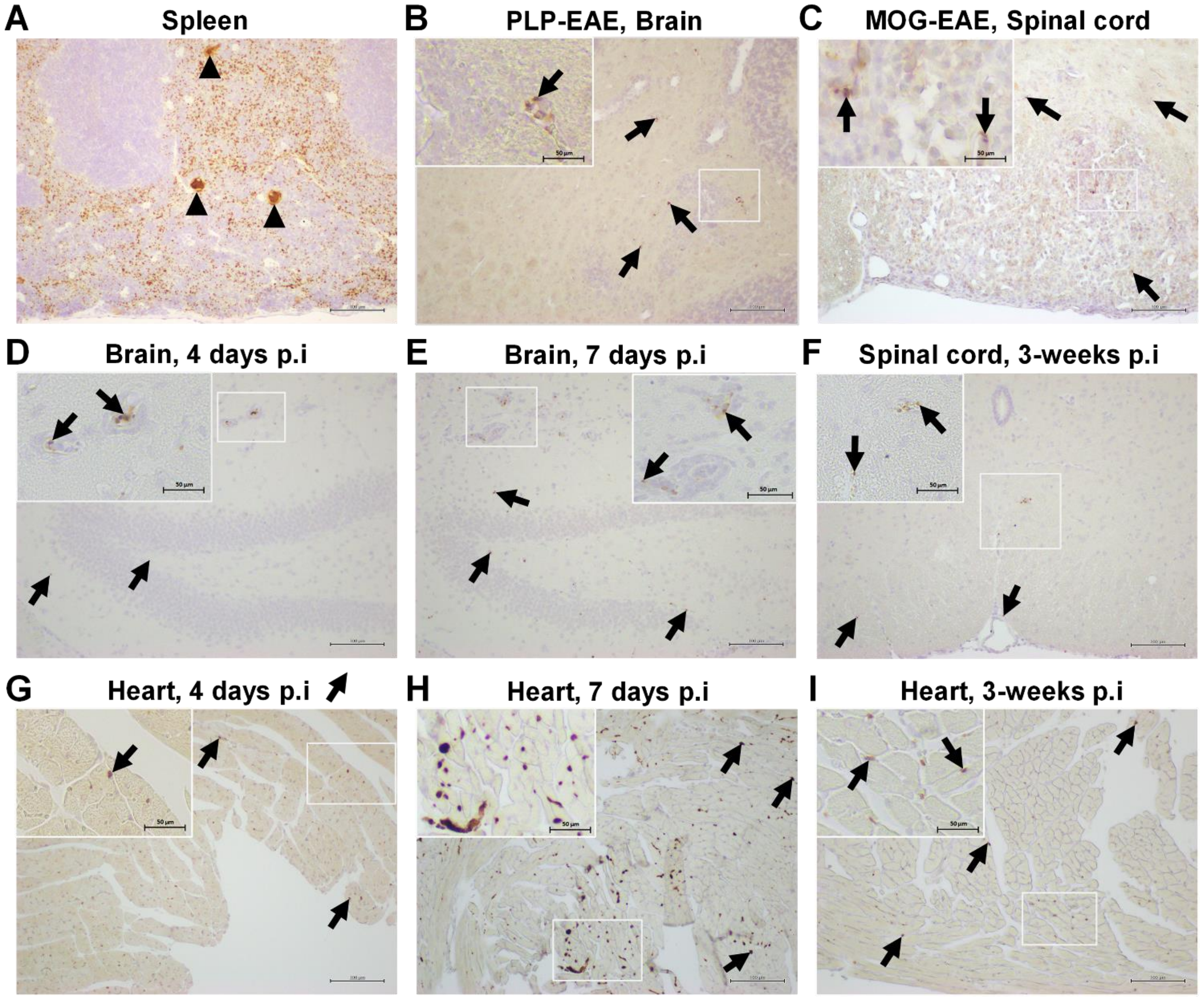

Supplemental Figure S6. Platelet detection in the central nervous system (CNS) and heart of TMEV infection and experimental autoimmune encephalomyelitis (EAE) induced by myelin proteolipid protein (PLP) $_{139-151}$ peptide, or myelin oligodendrocyte glycoprotein (MOG) $_{92-106}$ peptide. **(A-I)** We conducted immunohistochemistry against the platelet glycoprotein Ibα (GPIbα/CD42b, **A, B** and **D-I**) or platelet glycoprotein IIIa (GPIIIa/CD61/integrin β3, **C**), using the spleen, brain, spinal cord, and heart sections. **(A)** In the spleen, we used platelet-producing megakaryocytes (arrowheads) as a positive control; platelets were also detected in the red pulp. **(B, C)** We detected a small number of platelets adjacent to inflammatory demyelinating lesions in PLP-induced EAE and MOG-induced EAE. **(D-F)** In the CNS of TMEV infection, we detected a small number of platelets (arrows) sporadically, attached to the luminal side of vascular endothelia in the inflammatory brain lesions 4 and 7 days p.i. and a much fewer platelets attached to the vessels in the spinal cord 3-weeks p.i. **(G-I)** In the heart of TMEV infection, we detected a higher number of platelets (arrows) than the CNS; platelets were localized diffusely, irrelevant to cardiac lesions. Platelet accumulation in

the heart was more substantial 7 days p.i than 4 days and 3 weeks p.i. Five to six mice per time point. **(B)**, cerebellum; **(C, F)**, spinal cord; and **(D, E)**, hippocampus. Scale bar: **(A-I)** 100 μm; and inset, 50 μm.

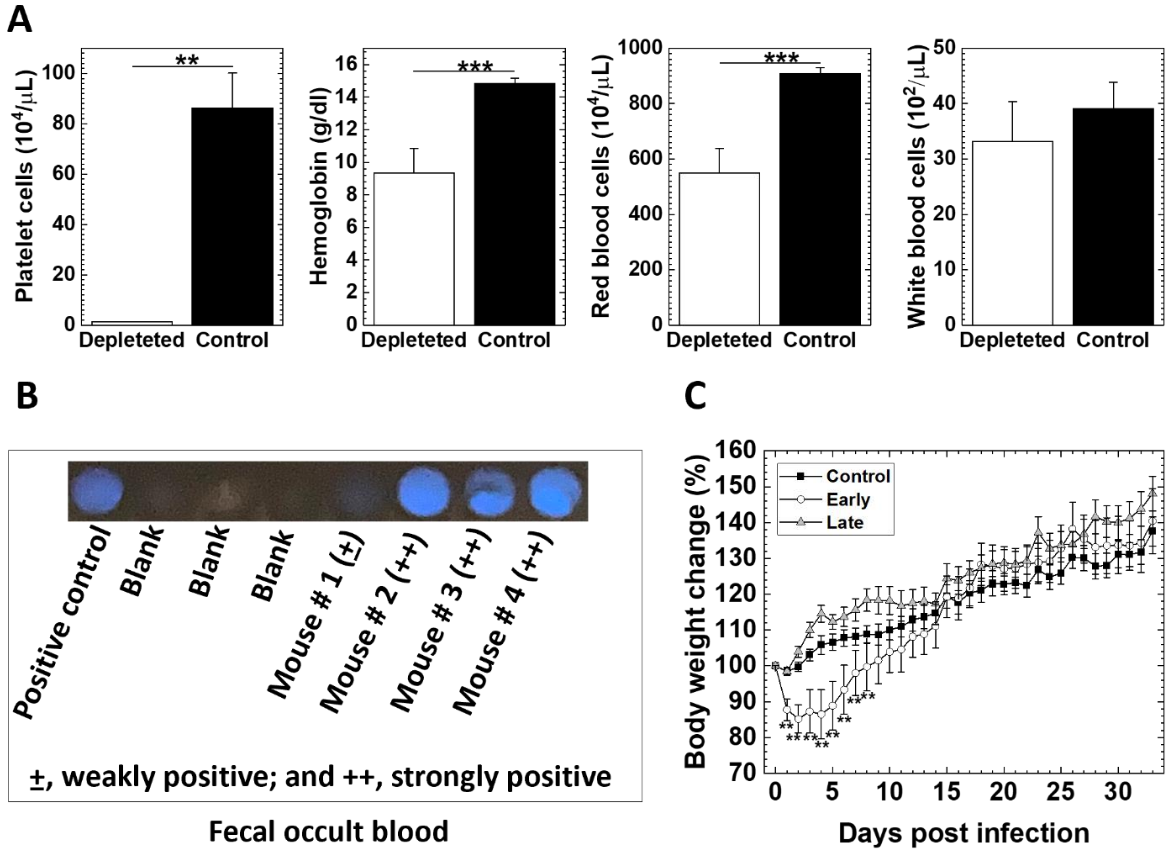

Supplemental Figure S7. Effects of platelet-depletion by antibody against GPIbα in mice. **(A)** We injected anti-GPIbα antibody (open bar, Depleted group) or control rat IgG (closed bar, Control group). We collected blood samples using a 5-mL tube containing 10 μL of 50 μg/mL EDTA-2Na by submandibular bleeding as well as fecal samples using a 1.8-mL cryotube, 4 days after the antibody injection. We counted the number of peripheral blood cells using Sysmex XT-1800i (Sysmex Corporation, Kobe, Japan) and found reductions in the platelets (98.6%), hemoglobin (37.2%), red blood cells (39.6%) and white blood cells (15.2%). There were statistical differences in platelets, hemoglobin, and red blood cells (**$P < 0.01$, ***$P < 0.001$, Student's *t*-test), but not in white blood cells, between the two groups. Four mice per group. Results are the mean + SEM. **(B)** Using the Luminol Reaction Kit® (Wako Pure Chemical, Osaka, Japan), we mixed a portion of feces with a luminol solution using a 96-well plate and observed in dark field. We detected fecal occult blood (FOB) in platelet-depleted mice: mouse # 1-4. The fecal samples from mouse # 2-4 were strongly positive; the FOB-positive feces generated strong blue-white luminescence. On the other hand, the fecal sample from mouse # 1 was weakly positive and generated weak blue-white luminescence. A hemoglobin solution (10 μg/mL) was used a positive control. **(C)** Body weight changes in TMEV-infected mice among the Control (■), Early (○), and Late (▲) groups. TMEV-infected mice were treated with anti-GPIba antibody twice days 0 and 5 (Early) or days 18 and 22 p.i (Late); TMEV-infected control mice (Control) were given the control antibody. After TMEV infection, we monitored body weight changes daily. In the first 8 days, the Early group had significant body weight loss (**$P < 0.01$, ANOVA), compared with the Control and Late groups. There were no statistical differences

between the Control and Late groups. Each group was composed of five to 14 mice. Results are the mean ± SEM.

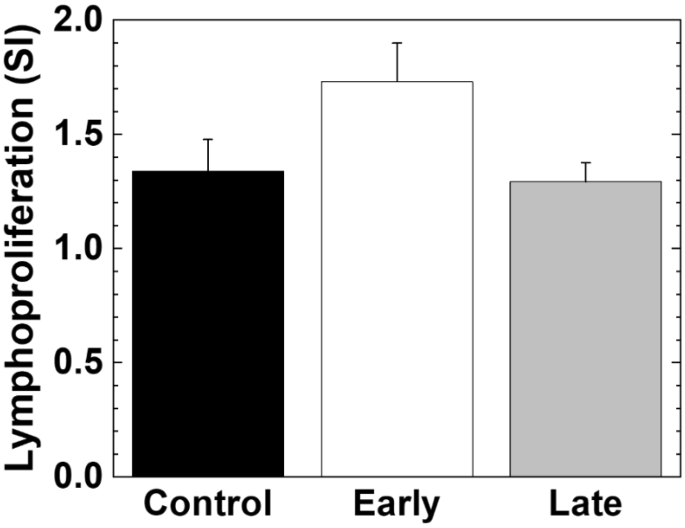

Supplemental Figure S8. Anti-viral lymphoproliferative responses in TMEV-infected mice treated with a rat IgG control antibody (Control) or a platelet-specific GPIbα (CD42b) depletion antibody during the early (Early) or late (Late) time point in TMEV infection. We determined the levels of lymphoproliferative responses to TMEV 35 days p.i. Splenic mononuclear cells were isolated from the Control, Early, and Late groups and stimulated with 1 µg of purified TMEV antigens. There were no statistical differences in the lymphoproliferative responses to the viral antigens among the three groups. The levels of anti-viral lymphoproliferation were determined by a Cell Counting Kit-8 reagent and were expressed as stimulation indexes (SI, experimental absorbance/control absorbance). Results are the mean + SEM from two to four pools of the spleens from two to three mice per group.

Gene symbol	Gene name	Fold change	*P*
Platelet-related genes upregulated in blood of TMEV-infected mice**			
Ppbp	pro-platelet basic protein	13.4	0.001
Pf4	platelet factor 4 (CXLC4)	9.6	0.002
Gp9	platelet glycoprotein IX (GP9, CD42a)	7.1	0.003
P2ry12	purinergic receptor P2Y, G-protein coupled 12	6.4	0.004
Mmrn1	multimerin 1	3.5	0.010
Pecam1	platelet endothelial cell adhesion molecule 1 (PECAM-1, CD31)	2.3	0.010
Mpig6b	megakaryocyte and platelet inhibitory receptor G6b (G6b-B)	2.1	0.031
Platelet-related genes upregulated in the spleen of SJL/J mice with progressive EAE***			
F13a1	coagulation factor XIII, A1 subunit	4.5	0.001
Pdgfra	platelet derived growth factor receptor (PDGF-R), alpha polypeptide	3.4	0.000
F5	coagulation factor V	3.1	0.000
Selp	p-selectin (CD62P)	2.8	0.000
Mmrn1	multimerin 1	2.7	0.001
Mpig6b	megakaryocyte and platelet inhibitory receptor G6b (G6b-B)	2.5	0.000
Pf4	platelet factor 4 (CXLC4)	2.3	0.000
Pla2g7	phospholipase A2, group VII (platelet-activating factor acetylhydrolase, plasma)	2.3	0.000
Gp5	platelet glycoprotein V (GP5, CD42d)	2.2	0.003
Gp1ba	platelet glycoprotein Ib, alpha polypeptide (CD42b)	2.2	0.004
F2rl2	coagulation factor II thrombin receptor like 2	2.1	0.004
Platelet-related genes upregulated in the spleen of A.SW mice with progressive EAE***			
Pdgfra	platelet derived growth factor receptor (PDGF-R), alpha polypeptide	2.9	0.007
Pla2g7	phospholipase A2, group VII (platelet-activating factor acetylhydrolase, plasma)	1.8	0.008
Pear1	platelet endothelial aggregation receptor 1	1.6	0.032
Pdgfrb	platelet derived growth factor receptor (PDGF-R), beta polypeptide	1.6	0.013
Pfkp	phosphofructokinase, platelet	1.3	0.010

Supplemental Table S1. Upregulations of platelet-related genes in TMEV infection and EAE*

*Upregulated genes with statistically significances ($P < 0.05$) in mice infected with Theiler's murine encephalomyelitis virus (TMEV) or mice induced with experimental autoimmune encephalomyelitis (EAE), compared with control mice. Three genes commonly upregulated in TMEV infection and the SJL/J EAE model are highlighted in yellow. Two genes commonly upregulated in the SJL/J EAE model and the A.SW EAE model are highlighted in pink.
**Blood transcriptome data from mice infected with TMEV 2 month post-infection (Omura et al., *Circ Cardiovasc Genet*, 2014, Omura and Tsunoda, *Pharma Medica*, 2021. Vol 38: 67-71).
***We induced EAE in SJL/J and A.SW mice by sensitization with the myelin oligodendrocyte glycoprotein (MOG)92−106 peptide. One day prior to MOG sensitization, SJL/J mice were also injected with curdlan (Omura et al., *Front Immunol*, 2019. Mehmood A et al., *Int J Exp Pathol*, 2023).
